# An Updated Digital Approach to Regional Anesthesia: A Pilot Study on Computer-Guided Maxillary Nerve Block via the Greater Palatine Canal

**DOI:** 10.3390/dj13110521

**Published:** 2025-11-06

**Authors:** Ioannis Fotopoulos, Anastasia Fardi, Vasileios Zisis, Athanasios Poulopoulos, Nikolaos Dabarakis, Theodoros Lillis

**Affiliations:** 1 Department of Dentoalveolar Surgery, Surgical Implantology and Radiology, School of Dentistry, Aristotle University of Thessaloniki, 54124 Thessaloniki, Greece; 2 Department of Oral Medicine/Pathology, School of Dentistry, Aristotle University of Thessaloniki, 54124 Thessaloniki, Greece; 3 Department of Dentistry (Oral Medicine-Oral Pathology), School of Dentistry, European University, Diogenous Street 6, 2404 Nicosia, Cyprus

**Keywords:** maxillary nerve block, greater palatine canal, computer-guided anesthesia, cone beam computed tomography (CBCT), regional dental anesthesia

## Abstract

**Objectives:** Maxillary nerve block via the greater palatine canal (GPC) offers the potential for profound regional anesthesia of the maxilla but remains underutilized due to anatomical variability and technical complexity. The aim of this study was to explore the clinical feasibility, accuracy, and anesthetic effectiveness of a computer-guided approach by using CBCT-based surgical guides to access the pterygopalatine fossa via the GPC. **Methods:** Thirty-one patients underwent the procedure with patient-specific guides designed from cone-beam computerized tomography (CBCT) and intraoral scans. A 27G needle was directed through the guide to deliver 1.8 mL of 2% lidocaine with epinephrine 1:80.000. Pulpal anesthesia was assessed via electric pulp testing (EPT), and soft tissue anesthesia via pressure algometry at predefined oral and facial sites. Success was defined as absence of EPT response at maximum output and pressure pain threshold ≥ 700 g. To assess variations in anesthetic efficacy among multiple related groups, Cochran’s Q test and McNemar’s test were employed. **Results:** Successful needle placement was achieved in 30 out of 31 patients (96.7%) using the computer-guided approach, with a mean of 1.45 insertion attempts per case. Complete palatal soft tissue anesthesia was achieved in all subjects across the tested sites (100%). Pulpal anesthesia was most effective in posterior teeth, with success rates of 96.7% for first molars and 93.3% for first premolars, while the central incisor showed a reduced success rate of 50%. Transient visual disturbances occurred in three patients (10%), with no other adverse effects reported. **Conclusions:** These findings support the use of computer-guided GPC block as a method for achieving maxillary nerve anesthesia. Although anesthetic spread to anterior and buccal regions was limited, the technique demonstrated consistent effectiveness in the posterior maxilla, highlighting its potential utility in complex dental and surgical interventions requiring deep and long-lasting regional anesthesia.

## 1. Introduction

Maxillary nerve block anesthesia via the greater palatine canal (GPC) is a valuable yet underutilized technique for achieving profound regional anesthesia of the maxilla. It targets the second division of the trigeminal nerve (V2) and provides effective analgesia for a wide range of anatomical structures, including the corresponding maxillary teeth, buccal and palatal mucosa, nasal cavity, maxillary sinus, and facial skin regions such as the lateral nose, infraorbital area, and upper lip [1,2,3]. Despite its wide-reaching anesthetic potential, the GPC approach is not routinely used in daily clinical practice due to anatomical complexity and the technical skill it demands [4,5,6,7]. Anatomical studies have highlighted substantial variation in the trajectory, length, and curvature of the greater palatine canal, which can complicate needle guidance and increase the risk of complications such as inadequate anesthesia, vascular damage, or injury to adjacent neural structures [8,9,10,11,12].

In parallel, digital technologies have revolutionized dental and surgical practice. The use of cone-beam computed tomography (CBCT), computer-aided design (CAD), and three-dimensional (3D) printing has facilitated the development of surgical guides with high anatomical fidelity [13,14]. These guides are now widely used in implantology and are increasingly adopted in applications such as sinus surgery, endodontics, and orthodontics [15,16]. Notably, recent case reports have demonstrated the successful use of CAD/CAM-guided surgical templates for the delivery of maxillary nerve block anesthesia via the GPC, showing improved accuracy and safety [17,18]. The guides, based on CBCT data and intraoral scans, were used to control the depth and angle of needle insertion, minimizing the risk of deviation and enhancing clinical predictability [17,18]. However, surgical guides cannot guarantee 100% accuracy, since potential errors may arise from CBCT acquisition, digital planning, or 3D printing. In the present study, our aim was to assess feasibility and safety, while acknowledging these inherent limitations. Future randomized controlled studies with direct comparison to conventional freehand techniques are necessary to establish the relative clinical benefits.

The precise assessment of local anesthesia success remains a challenge in clinical settings. Conventional techniques often rely on subjective feedback, such as patient-reported numbness, typically evaluated by applying constant force to the anesthetized soft tissue using a dental explorer or a tweezer [19,20]. However, these methods lack standardization and reproducibility, limiting their reliability in both clinical and research contexts. In contrast, objective tools like electric pulp testing (EPT) and pressure algometry have emerged as valuable instruments to measure pulpal and soft tissue anesthesia, respectively [21,22]. These techniques enable quantifiable assessment of anesthetic depth and distribution and may offer greater diagnostic reliability when evaluating local anesthesia techniques including computer-guided anesthesia [23,24,25].

The aim of this study is to evaluate the applicability of maxillary nerve block anesthesia administered via the pterygopalatine canal by using computer-designed surgical guides. These guides are intended to direct the needle with precision through the greater palatine foramen and into the canal, thereby improving needle trajectory, minimizing procedural risk, and enhancing the reproducibility of the technique in clinical practice. Utilizing CBCT-based digital planning software in combination with 3D-printed templates, this clinical investigation seeks to assess the accuracy, safety, and anesthetic efficacy of the technique in patients undergoing maxillary surgical procedures.

## 2. Materials and Methods

### 2.1. Study Design and Setting

This prospective clinical study was conducted at the Department of Dentoalveolar Surgery, Implantology and Radiology, School of Dentistry, Aristotle University of Thessaloniki (the study was conducted from 1 October 2023 to 15 July 2024). Ethical approval was granted by the Institutional Ethics Committee (Approval No. 20/29-06-2023), and all procedures were performed in accordance with the Declaration of Helsinki. Written informed consent was obtained from all participants.

### 2.2. Volunteer Selection

Thirty-one patients (13 men and 18 women; mean age, 24.5 years) were initially enrolled in the study. Participants were recruited among individuals who had undergone CBCT imaging of the maxilla as part of preoperative planning for scheduled surgical procedures, such as implant placement or extraction of impacted teeth. Following completion of their planned surgical intervention, patients were invited to voluntarily participate in a separate study appointment dedicated to the anesthesia evaluation protocol. The appointment for anesthesia testing was scheduled at least one month after the initial surgical procedure to allow for adequate healing and to avoid confounding factors related to postoperative changes. Eligible patients were required to present an intact maxillary central incisor, first premolar, and first molar (free of caries, restorations, trauma history, sensitivity, or periodontal disease) in the quadrant of interest. Exclusion criteria included the use of medications that could interfere with anesthetic assessment, known allergies to local anesthetics, pregnancy or lactation, and the presence of active pathological lesions at the intended injection site.

### 2.3. Guide Design and Fabrication

A customized surgical guide was fabricated for each patient using a combination of CBCT imaging (NewTom 5G XL^®^, Cefla Group, Imola, Italy) and intraoral scanning (TRIOS 5^®^ intraoral scanner, 3Shape, Copenhagen, Denmark). Digital design was performed using Implant Studio^®^ (3Shape, Copenhagen, Denmark). Each guide was designed to include a metallic sleeve positioned along the planned trajectory of the greater palatine canal, ensuring precise needle insertion with controlled depth and angulation. Guides were fabricated using the Original Prusa Medical One^®^ 3D printer (Prusa Research, Prague, Czech Republic) with 3DShining Surgical Guide Resin (3DShining Tech Co., Ltd., Hangzhou, China), a certified biocompatible Class I medical material suitable for intraoral use.

### 2.4. Pulp Tissue Anesthesia Assessment

Pulp vitality was assessed using a digital electric pulp tester (model ADS-T1, Foshan Adelson Medical Devices Co., Ltd., Foshan, China). Testing was performed on the maxillary central incisor (PCI), first premolar (PFP), and first molar (PFM) within the anesthetized quadrant. The pulp tester electrode was applied to the buccal surface of each tooth, using a thin layer of toothpaste as a conducting medium. The output intensity of the device gradually increased, and the subject was instructed to raise his or her hand immediately upon feeling any sensation. The value at which the subject responded was recorded. Each tooth was initially tested at baseline and at subsequent time intervals after anesthesia administration (5, 10, 20, 30, 45, 60, 90, 120, and 180 min). Pulpal anesthesia was defined as the absence of response even when the stimulus reached the maximum output of 80 on the device scale.

### 2.5. Soft Tissue Anesthesia Assessment

The anesthetized areas were evaluated by measuring pressure pain thresholds (PPT) at nine predefined sites. These included the buccal mucosa adjacent to the maxillary central incisor (BMCI), first premolar (BMFP), first molar (BMFM), the palatal mucosa adjacent to the central incisor (PMCI), first premolar (PMFP), and first molar (PMFM), the skin at the infraorbital region (SIR), the lateral side of the nose (SLSN), and the upper lip (SUL). A digital force meter with a rounded 2 mm probe (model AMF-10, ALIYIQI; Wenzhou Tripod Instrument Manufacturing Co., Ltd., Wenzhou, China) was used for all measurements. The device applied gradually increasing perpendicular pressure to the site until the subject indicated pain perception. The applied force (grams) was displayed digitally and recorded as the pressure pain threshold (PPT). A maximum load of 700 g was predetermined to avoid tissue injury [26]. Each site was tested at baseline and at subsequent intervals after administration of the maxillary nerve block (5, 10, 20, 30, 45, 60, 90, 120, and 180 min). Soft tissue anesthesia at a given site was defined as the ability to tolerate the maximum force (700 g) without reporting pain.

### 2.6. Maxillary Nerve Anesthesia Protocol

**Figure 1:**
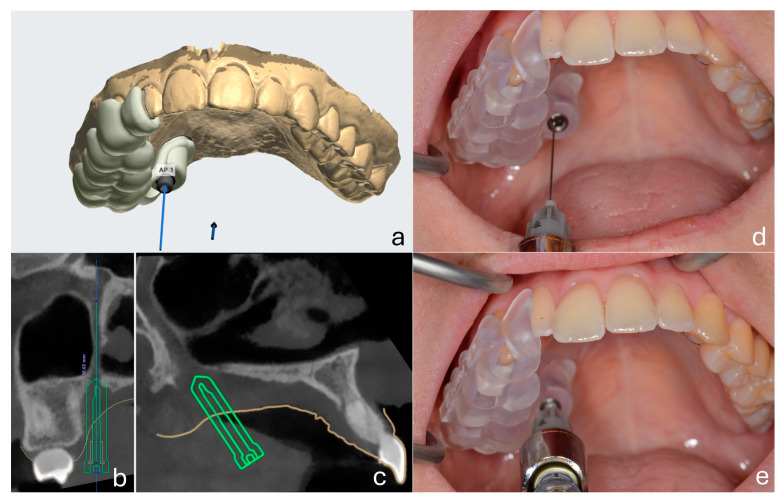
Computer-guided maxillary nerve block via the greater palatine canal using a customized surgical guide. (**a**) Virtual planning of the guide and insertion trajectory on maxillary digital cast. (**b**,**c**) CBCT sections showing the planned path of needle insertion into the pterygopalatine canal in relation to surrounding anatomy. (**d**,**e**) Clinical application of the 3D-printed guide with the needle inserted through the metallic sleeve during local anesthetic administration.

After baseline testing, the customized surgical guide was positioned intraorally. A 27G long dental needle (Septoject^®^, Septodont, Saint-Maur-des-Fossés, France) was inserted through the guide and advanced into the greater palatine canal under controlled depth and angulation to perform the maxillary nerve block. The metallic sleeve of the guide functioned as both an angulation stabilizer and a depth limiter. The hub of the needle contacted the sleeve ring, providing a physical stop and ensuring standardized penetration depth across all patients. The anesthetic solution administered was 2% lidocaine with 1:80.000 epinephrine (Lignospan Special^®^, Septodont, Saint-Maur-des-Fossés, France), with a total volume of 1.8 mL delivered per patient. All nerve block procedures and measurements were performed by the same experienced oral surgeon to ensure consistency and minimize operator-related variability. The technique for performing the nerve block was based on the method described by Malamed [27] (Figure 1).

### 2.7. Statistical Analysis

Descriptive statistics (mean ± standard deviation) were calculated for all continuous variables, including pressure pain thresholds (PPT) and electric pulp tester (EPT) readings at each anatomical site and time point. The temporal progression of anesthesia was analyzed for each site individually. Cochran’s Q test—a non-parametric test suitable for analyzing differences in dichotomous outcomes measured across three or more related conditions—was employed to compare anesthetic efficacy across multiple related groups. After a significant Cochran’s Q test, McNemar’s test was used for pairwise comparisons, with Bonferroni correction for multiple comparisons. The level of statistical significance was set at *p* < 0.05 unless adjusted as needed. All analyses were performed using SPSS software (Version 27.0; IBM Corp., Armonk, NY, USA).

## 3. Results

During the procedure, one patient was excluded from further evaluation due to technical failure: inability to advance the needle through the surgical guide into the greater palatine canal after several attempts. Consequently, data from thirty patients were analyzed. In all thirty patients, the needle was successfully directed through the customized surgical guide into the greater palatine canal without deviation and without the need for needle bending. The mean number of insertion attempts per guide was 1.45.

Anesthetic success in the palatal mucosa was uniformly high across all test locations (Table 1). All thirty patients (100%) exhibited successful anesthesia in the palatal mucosa adjacent to the central incisor (PMCI), first premolar (PMFP), and first molar (PMFM). Onset of anesthesia occurred rapidly on palatal mucosa, with mean values ranging from 5.0 min (PMFM) to 5.8 min (PMCI), and durations ranging from 73.2 ± 32.5 min to 114 ± 52.4 min.

In contrast, buccal mucosal anesthesia demonstrated moderate to low success rates. The posterior region adjacent to the first molar (BMFM) showed the highest buccal success (40%), followed by the first premolar (BMFP, 23.3%) (middle region) and central incisor (BMCI, 6.7%) (anterior region). The onset times were longer than those of the palatal sites, and durations were notably shorter, especially in the anterior regions (BMCI duration: 10 ± 0 min). After applying the Bonferroni correction (adjusted alpha = 0.017), the comparison between the anterior and posterior region of buccal mucosa was statistically significant (*p* = 0.002), indicating a difference in efficacy. The comparisons between the anterior and middle (*p* = 0.063) and between the middle and posterior (*p* = 0.063) were not statistically significant.

Facial skin regions demonstrated minimal responsiveness to the anesthetic technique. No subject (0%) exhibited successful anesthesia in the infraorbital region (SIR). Limited success (6.7%) was observed at the lateral side of the nose (SLSN) and (3.3%) upper lip (SUL), both with brief onset and short anesthetic duration. There were no statistically significant differences in anesthetic efficacy among the three positions, χ^2^(2) = 3.0, *p* = 0.223.

**Figure 2:**
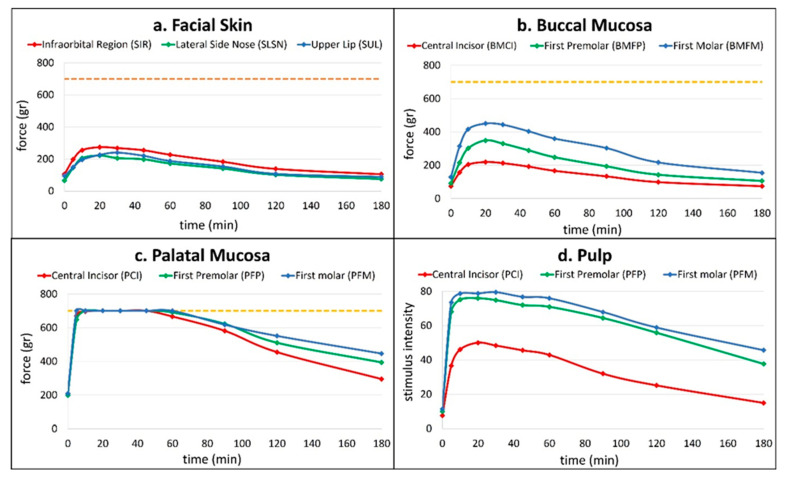
Time-dependent changes in anesthetic efficacy across facial, oral soft tissue, and pulpal sites following computer-guided maxillary nerve block via the greater palatine canal. Graphs depict the mean pressure pain threshold (PPT, in grams) over time at (**a**) facial skin sites (infraorbital region, lateral side of the nose, upper lip), (**b**) buccal mucosa adjacent to the central incisor, first premolar, and first molar, and (**c**) palatal mucosa adjacent to the same teeth. Graph (**d**) illustrates pulp sensibility responses (stimulus intensity) assessed by electric pulp testing (EPT). The horizontal dashed orange line at 700 g (**a**–**c**) represents the predefined threshold for successful soft tissue anesthesia.

Pulpal anesthesia was achieved most consistently in posterior teeth. The first molar (PFM) exhibited a 96.7% success rate with a mean onset of 5.5 ± 1.5 min and a duration of 114 ± 54.9 min. The first premolar (PFP) followed, with 93.3% success, onset at 6.3 ± 3.2 min, and a duration of 98.8 ± 48.2 min. The central incisor (PCI) demonstrated the lowest success rate at 50%, with delayed onset (9 ± 0 min) and shorter duration (67 ± 41.5 min) (Figure 2).

The comparisons between incisors and molars (*p* < 0.001), as well as between incisors and premolars (*p* < 0.001), were statistically significant, indicating differences in efficacy. In contrast, the comparison between premolars and molars (*p* = 1.000) was not statistically significant after the correction.

Three participants (10%) reported transient ophthalmologic disturbances following the injection. Specifically, two patients experienced brief episodes of blurred vision, and one reported temporary diplopia. These symptoms appeared within 5 to 10 min after administration and resolved spontaneously within 30 min without the need for intervention. No cases of prolonged visual disturbance, ptosis, or ocular pain were observed. All other patients remained asymptomatic, and no systemic adverse effects were recorded during or after the procedure.

## 4. Discussion

A computer-guided approach to maxillary nerve block via the greater palatine canal (GPC) was evaluated in terms of feasibility and safety. By utilizing patient-specific surgical guides, access to the pterygopalatine canal was generally achieved with precision, enabling accurate anesthetic delivery to the maxillary nerve. Previous clinical studies have shown that both the GPC and high tuberosity (HT) techniques for V2 anesthesia block can be comparably effective and technically manageable [2,6,28]. However, the GPC approach has been associated with more reliable anesthetic outcomes in the posterior regions, especially when anatomical landmarks are accurately followed [1,3,29]. In the present study, the combination of digitally planned needle trajectory and controlled depth of penetration allowed for reproducible targeting of the pterygopalatine fossa, supporting the potential for broader regional anesthesia.

The anesthesia distribution observed was consistent with previous findings, demonstrating high efficacy in posterior regions and reduced anesthetic effect in anterior and facial areas [1,2,3,6]. This pattern may be explained by two key mechanisms. First, according to the core theory of nerve fiber organization, pulpal fibers from anterior teeth are located centrally within the nerve and may be less accessible to anesthetic agents deposited at more peripheral sites. This fascicular arrangement, described by Cho et al., can explain the relative resistance of anterior pulps to regional block [26]. Second, the posterior palatal and buccal soft tissues are innervated by branches such as the greater palatine and posterior superior alveolar nerves that emerge in close proximity to the site of injection. In contrast, anterior regions are innervated by more distal branches, including the nasopalatine, anterior superior alveolar, and infraorbital nerves, which are less likely to be adequately anesthetized due to their distance from the injection site. These anatomical features, supported by both anatomical and clinical studies likely account for the observed regional differences in anesthetic success [6,10,19]. Notably, in this study, complete anesthesia was achieved in over 90% of posterior palatal and buccal sites within 20 min, compared to fewer than 40% of anterior sites.

Considerable anatomical variability in the course and angulation of the greater palatine canal has been documented, posing challenges for freehand needle placement and potentially compromising anesthetic efficacy [3,4,19,30]. These findings support the integration of CBCT-assisted planning to improve trajectory control and minimize procedural risks. The guided approach used in our study, based on individualized digital planning, appeared to mitigate these limitations, enhancing both accuracy and consistency. Similar advantages have been reported by Jamjoom et al. and Jundt et al., who demonstrated improved needle control and patient safety using computer-guided anesthesia protocols [17,18]. In our sample, this method enabled effective canal access with a mean of 1.45 insertion attempts and only one recorded technical failure. As previous studies rarely quantified insertion attempts or reported failure rates, our findings suggest that computer-guided techniques may offer greater standardization and reproducibility in clinical practice.

An additional advantage of the computer-guided technique relates to the required volume of local anesthetic. In the present study, a total volume of 1.8 mL of 2% lidocaine with epinephrine 1:80.000 was sufficient to achieve consistent anesthesia in the target quadrant. This finding aligns with previous reports, where a single injection of 1.8 mL proved adequate for maxillary surgical procedures [6,7,19,27]. Similarly, Jundt et al. emphasized that personalized 3D-guided techniques allow for optimized needle placement and may help reduce the amount of local anesthetic required [18]. These observations support the notion that accurate needle guidance and trajectory control can minimize the need for higher anesthetic volumes, reducing systemic exposure and procedural variability.

Baseline tissue sensitivity, as assessed via pressure pain threshold (PPT), may also influence both the subjective perception and objective efficacy of anesthesia. In this study, we used 700 g as the cut-off point to define successful soft tissue anesthesia, in line with previous research [26]. We employed both pressure algometry and electric pulp testing (EPT) to objectively evaluate the depth and extent of anesthesia. However, we noted differences between soft tissue and pulpal anesthesia responses, especially in the anterior regions, confirming earlier findings that soft tissue numbness does not necessarily indicate pulpal anesthesia [6,26]. This discrepancy is likely due to the deeper location of pulpal nerve fibers, which may not be equally affected by anesthetic diffusion.

Despite the overall favorable safety profile, three cases (10%) of transient ophthalmologic disturbances were recorded. Specifically, two patients reported brief blurred vision, and one experienced temporary diplopia. These effects appeared within the first 10 min post-injection and resolved spontaneously within 30 min without requiring medical intervention. This rate is markedly lower than the 35.6% incidence of diplopia reported by Sved et al. in a large cohort treated with a freehand GPC block [31]. These visual effects are likely related to anesthetic diffusion toward the orbit through anatomical communications such as the inferior orbital fissure [4,31]. No serious adverse events, including prolonged paresthesia, soft tissue injury, or infection, were observed. Notably, no cases of palatine nerve injury or intravascular injection were observed, which are complications previously reported in 1% and 7.9% of freehand cases, respectively [2,4,31]. These outcomes reinforce the safety, precision, and clinical utility of the computer-guided approach.

This study has several limitations. First, no control group was included, as the primary aim was to assess the feasibility and anesthetic efficacy of a computer-guided approach. Consequently, a direct comparison of the anesthetic success rates of this method with the freehand method could not be evaluated. Second, no a priori sample size calculation was performed; however, the number of participants is comparable to similar studies in the field. Moreover, since the selection of participants in the present study was not randomized, the results cannot be directly generalized to the broader patient population. Finally, aspects such as cost-effectiveness, patient comfort, and practical integration of the method into routine practice were not systematically evaluated and should be addressed in future research. Further randomized controlled trials with larger samples are required to confirm these preliminary observations and to better define the clinical role of computer-guided maxillary nerve block.

## 5. Conclusions

In conclusion, the use of a computer-guided surgical guide for maxillary nerve block via the GPC provides accurate, reproducible, and safe access to the maxillary nerve. While anterior and buccal tissues may show reduced anesthetic response due to anatomical and physiological factors, the approach holds considerable promise in complex dental and surgical procedures where profound and consistent anesthesia is essential. This technique would have been much more significant if it had shown better success rates for pulpal anesthesia when the computer was used.

## Figures and Tables

**Table 1:** Success rate, onset time, and duration of anesthesia at selected facial, mucosal, and pulpal sites (n = 30).

Site	Success Rate (%)	Onset (min) (SD)	Duration (min) (SD)
**Facial Skin**			
Infraorbital Region (SIR)	0/30 (0%)	–	–
Lateral Side of Nose (SLSN)	2/30 (6.7%)	10 ± 0	15 ± 7.1
Upper Lip (SUL)	1/30 (3.3%)	20	10
**Buccal Mucosa**			
Central Incisor (BMCI)	2/30 (6.7%) *	15 ± 7.1	10 ± 0
First Premolar (BMFP)	7/30 (23.3%)	14.3 ± 5.3	22.9 ± 7.6
First Molar (BMFM)	12/30 (40%) *	12.9 ± 7.8	33.8 ± 22.7
**Palatal Mucosa**			
Central Incisor (PMCI)	30/30 (100%)	5.8 ± 2.9	73.2 ± 32.5
First Premolar (PMFP)	30/30 (100%)	5.7 ± 1.7	100.3 ± 50.1
First Molar (PMFM)	30/30 (100%)	5 ± 0	114 ± 52.4
**Pulp**			
Central Incisor (PCI)	15/30 (50%) **	9 ± 0	67 ± 41.5
First Premolar (PFP)	28/30 (93.3%) **	6.3 ± 3.2	98.8 ± 48.2
First Molar (PFM)	29/30 (96.7%) **	5.5 ± 1.5	114 ± 54.9

**Footnotes:** SIR: Skin Infraorbital Region; SLSN: Skin Lateral Side of Nose; SUL: Skin Upper Lip; BMCI: Buccal Mucosa Central Incisor; BMFP: Buccal Mucosa First Premolar; BMFM: Buccal Mucosa First Molar; PMCI: Palatal Mucosa Central Incisor; PMFP: Palatal Mucosa First Premolar; PMFM: Palatal Mucosa First Molar; PCI: Pulp Central Incisor; PFP: Pulp First Premolar; PFM: Pulp First Molar; SD = standard deviation; min = minutes; * *p* < 0.05; ** *p* < 0.001.

## Data Availability

The original contributions presented in this study are included in the article. Further inquiries can be directed to the corresponding authors.

## References

[1] Aoun G., Zaarour I., Sokhn S., Nasseh I. (2015). Maxillary nerve block via the greater palatine canal: An old technique revisited. J. Int. Soc. Prev. Community Dent..

[2] Broering R., Reader A., Drum M., Nusstein J., Beck M. (2009). A prospective, randomized comparison of the anesthetic efficacy of the greater palatine and high tuberosity second division nerve blocks. J. Endod..

[3] Hawkins J.M., Isen D. (1998). Maxillary nerve block: The pterygopalatine canal approach. J. Calif. Dent. Assoc..

[4] Sharma N., Varshney R., Ray S. (2014). Anatomic and anaesthetic considerations of greater palatine nerve block in Indian population. Saudi J. Med. Med. Sci..

[5] Mahesh A., Rajesh S. (2021). A study of greater palatine foramen and its importance in the application of maxillary nerve block in South Indian population. Int. J. Anat. Res..

[6] Bacci C., Ferrario S., Sivolella S., Menozzi G., Bartorelli L., Grossi G., Zanett G. (2018). Maxillary nerve block: A comparison between the greater palatine canal and high tuberosity approaches. Ital. J. Dent. Med..

[7] Cohn S.A. (1986). The advantages of the greater palatine foramen block technique. J. Endod..

[8] Kim D.W., Tempski J., Surma J., Ratusznik J., Raputa W., Świerczek I., Pękala J.R., Tomaszewska I.M. (2023). Anatomy of the greater palatine foramen and canal and their clinical significance in relation to the greater palatine artery: A systematic review and meta-analysis. Surg. Radiol. Anat..

[9] El-Anwar M.W., Eldib D.B., Haggag M.S., Almolla R.M., Elbary M.E.-S.A., Abdelaal T.M., Khazbak A. (2021). Pterygopalatine fossa: A computed tomography analysis and classification. Pan Arab. J. Rhinol..

[10] Aoun G., Nasseh I., Sokhn S. (2016). Radio-anatomical Study of the Greater Palatine Canal and the Pterygopalatine Fossa in a Lebanese Population: A Consideration for Maxillary Nerve Block. J. Clin. Imaging Sci..

[11] Machado A., Simmen D., Schuknecht B., Briner H.R. (2022). Greater palatine canal: Computed tomography-based anatomic analysis and clinical significance for the sinus and skull base surgeon. Ear Nose Throat J..

[12] Chrcanovic B.R., Custódio A.L.N. (2010). Anatomical variation in the position of the greater palatine foramen. J. Oral Sci..

[13] D’haese J., Ackhurst J., Wismeijer D., De Bruyn H., Tahmaseb A. (2017). Current state of the art of computer-guided implant surgery. Periodontology 2000.

[14] Fotopoulos I., Lillis T., Panagiotidou E., Kapagiannidis I., Nazaroglou I., Dabarakis N. (2022). Accuracy of dental implant placement with 3D-printed surgical templates by using Implant Studio and MGUIDE. An observational study. Int. J. Comput. Dent..

[15] Ayaş B.D., Çiçekcibaşı A.E., Gökşan A.S., Açar G., Aydoğdu D. (2024). Clinically relevant morphometric analysis of pterygopalatine fossa and its volumetric relationship with adjacent paranasal sinuses: A CT-based study. Oral Radiol..

[16] Kamel A.A., Harhash K., Abd Al-lateef M. (2022). Efficacy of pterygopalatine fossa injection in reducing intra-operative bleeding. Egypt J. Otolaryngol..

[17] Jamjoom F.Z., Doliveux S., Rousson D.D., Friedland B., Hamilton A. (2019). A modified implant surgical guide for the administration of maxillary nerve block anesthesia intraorally via the greater palatine foramen: Case report. Int. J. Oral Maxillofac. Implants..

[18] Jundt J.S., Chow C.C., Couey M. (2020). Computed tomography-guided 3D printed patient-specific regional anesthesia. J. Dent. Anesth. Pain. Med..

[19] Wong J.D., Sved A.M. (1991). Maxillary nerve block anaesthesia via the greater palatine canal: A modified technique and case reports. Aust. Dent. J..

[20] Baddour H.M., Hubbard A.M., Tilson H.B. (1979). Maxillary nerve block used prior to awake nasal intubation. Anesth. Prog..

[21] Manta K., Dabarakis N., Lillis T., Fotopoulos I. (2023). Anesthetic efficacy of buffered 4% articaine for mandibular first molar infiltration: A crossover clinical trial. J. Dent. Anesth. Pain Med..

[22] Yekta-Michael S., Stein J.M., Marioth-Wirtz E. (2015). Evaluation of the anesthetic effect of epinephrine-free articaine and mepivacaine through quantitative sensory testing. Head Face Med..

[23] Costa Y., Castrillon E., Bonjardim L., Conti P.R., Baad-Hansen L., Svensson P. (2017). Effects of experimental pain and lidocaine on mechanical somatosensory profile and face perception. J. Oral Facial Pain Headache.

[24] Kothari S.F., Shimosaka M., Iida T., Komiyama O., Shibutani K., Svensson P., Baad-Hansen L. (2019). Quantitative and qualitative assessment of sensory changes induced by local anesthetics block of two different trigeminal nerve branches. Clin. Oral Investig..

[25] Ogimoto T., Ogawa T., Sumiyoshi K., Matsuka Y., Koyano K. (2002). Pressure–pain threshold determination in the oral mucosa: Validity and reliability. J. Oral Rehabil..

[26] Cho S.-Y., Choi W., Kim J., Kim S.-T., Kim H.-J., Jung I.-Y. (2018). Anesthetic efficacy of an inferior alveolar nerve block in soft tissue and correlation between soft tissue and pulpal anesthesia. Clin. Oral Investig..

[27] Malamed S.F. (2020). Handbook of Local Anesthesia.

[28] Sundar G.T.P., Shetty T.P., Bylapudi B., Shetty V., Castellino C., Rai A., Karikal A., Shetty P. (2020). Effectiveness of the greater palatine nerve block for anaesthetising anterior palate: A prospective study. J. Clin. Diagn. Res..

[29] Bhullar R.S., Batra R., Sandhu H. (2021). Mapping the extent of greater palatine nerve block: A clinical study. J. Adv. Med. Dent. Scie Res..

[30] Mahesh A.M., Assis F.P., Jose J.E., Rajesh S. (2023). Morphometric anatomical variations of greater palatine canal and its clinical implications: A dry human skull study. Int. J. Pharm. Clin. Res..

[31] Sved A.M., Wong J.D., Donkor P., Horan J., Rix L., Curtin J., Vickers R. (1992). Complications associated with maxillary nerve block anaesthesia via the greater palatine canal. Aust. Dent. J..

